# Complete Genome Sequence of *Porphyrobacter* sp. Strain CACIAM 03H1, a Proteobacterium Obtained from a Nonaxenic Culture of *Microcystis aeruginosa*

**DOI:** 10.1128/genomeA.01069-17

**Published:** 2017-10-12

**Authors:** Wendel de Oliveira Castro, Alex Ranieri Jerônimo Lima, Pablo Henrique Gonçalves Moraes, Andrei Santos Siqueira, Délia Cristina Figueira Aguiar, Anna Rafaella Ferreira Baraúna, Luisa Carício Martins, Hellen Thais Fuzii, Danielle Costa Carrara Couto, Regiane Silva Kawasaki Francês, Clayton Pereira Silva de Lima, João Lídio Silva Gonçalves Vianez-Júnior, Márcio Roberto Teixeira Nunes, Leonardo Teixeira Dall’Agnol, Evonnildo Costa Gonçalves

**Affiliations:** a Laboratório de Tecnologia Biomolecular, Instituto de Ciências Biológicas (ICB), Universidade Federal do Pará (UFPA), Belém, Pará, Brazil; b Laboratório de Patologia Clínica e Doenças Tropicais, Núcleo de Medicina Tropical, Universidade Federal do Pará, Belém, Pará, Brazil; c Laboratório de Computação Aplicada (LCA), UFPA, Campus de Ananindeua, Pará, Brazil; d Laboratório de Bioinformática e Computação de Alto Desempenho (LABIOCAD), Instituto de Ciências Exatas e Naturais (ICEN), UFPA, Belém, Pará, Brazil; e Centro de Inovações Tecnológicas (CIT), Instituto Evandro Chagas (IEC), Ananindeua, Pará, Brazil; f Universidade Federal do Maranhão (UFMA), Campus de Bacabal, Maranhão, Brazil

## Abstract

Here, we present a complete genome sequence and annotation of *Porphyrobacter* sp. strain CACIAM 03H1, which was obtained from a nonaxenic culture of *Microcystis aeruginosa*, a cyanobacterium isolated from an Amazonian freshwater environment.

## GENOME ANNOUNCEMENT

*Porphyrobacter* is one of the five genera affiliated with the family *Erythrobacteraceae*, which was proposed in 2005 based on phylogenetic studies of the *Alphaproteobacteria* class. *Erythrobacteraceae* members are Gram-negative, aerobic, rod-shaped or pleomorphic coccoid, and non-spore-forming bacteria. Furthermore, they are chemo-organotrophic and most often associated with freshwater environments (1).

Since the availability of genomic data from Amazonian proteobacteria is scarce, we recovered the complete genome of a *Porphyrobacter* sp., which was obtained from a culture of *Microcystis aeruginosa* sp. strain CACIAM 03 (BioSample SAMN05467875). This cyanobacterium was isolated from a water sample from the Tucuruí Hydroelectric Power station reservoir (3°50′04.9″S, 49°42′32.2″W) in Pará, Brazil.

After DNA extraction of the nonaxenic culture, two sequencing runs were performed on the GS FLX 454 (Roche Life Sciences) platform using nonpaired libraries, and one sequencing run was carried out on the Illumina MiSeq platform using a paired-end library with 150-bp read length. All the raw reads were quality filtered, with a minimum Phred score of 20. A coassembly of all reads was performed by the assembly software Newbler version 2.9 (2) (which was parameterized with a minimum overlap of 40 bp, minimum overlap identity of 90%, heterozygote mode, and extend low-depth overlap options on), producing 1,484 scaffolds.

MaxBin 2.2.1 (3) was used to bin the set of assembled scaffolds. To classify taxonomically the obtained bins, we performed a BLASTp search for each bin in the sequences containing hidden Markov models for essential genes identified by MaxBin 2.2.1 against the NCBI nonredundant database. The results were visualized on MegaN 5.11.3 (4).

The bin identified as *Porphyrobacter* contained 6 scaffolds with an *N*_50_ value of 1,078,724 bp, totaling 3.5 Mb. To obtain the complete genome of *Porphyrobacter* sp. strain CACIAM 03H1, we aligned the scaffolds with the *Porphyrobacter* sp. strain LM 6 genome using Mauve 2.4.0 (5). Then, a manual gap closure step was done mapping the reads to the sequence with Geneious R9 (6).

The complete genome sequence has 3.5 Mb, 105 single-copy marker genes (104 unique), and 67.6% GC content and was estimated to be 97.2% complete. An automatic annotation process using the NCBI Prokaryotic Genome Annotation Pipeline (PGAP) (7) identified 3,281 coding sequences (CDSs), 44 tRNAs, 3 rRNAs, and 4 noncoding RNAs (ncRNAs). Overall, the data presented here will improve genomic information about *Erythrobacteraceae* members. Furthermore, it should provide further insights with regard to aerobic anoxygenic phototrophic metabolism, physiological nature, and biotechnological purposes.

### Accession number(s).

This whole-genome shotgun project was deposited in DDBJ/EMBL/GenBank under the accession number CP021378. The version described in this paper is version CP021378.1.

## References

[1] Tonon LAC, Moreira APB, Thompson F 2014 The family *Erythrobacteraceae*, p 213–235. *In* Rosenberg E, DeLong EF, Lory S, Stackebrandt E, Thompson F, The prokaryotes: *Alphaproteobacteria* and *Betaproteobacteria*, 4th ed. Springer, New York, NY.

[2] Margulies M, Egholm M, Altman WE, Attiya S, Bader JS, Bemben LA, Berka J, Braverman MS, Chen YJ, Chen Z, Dewell SB, Du L, Fierro JM, Gomes XV, Godwin BC, He W, Helgesen S, Ho CH, Ho CH, Irzyk GP, Jando SC, Alenquer MLI, Jarvie TP, Jirage KB, Kim JB, Knight JR, Lanza JR, Leamon JH, Lefkowitz SM, Lei M, Li J, Lohman KL, Lu H, Makhijani VB, McDade KE, McKenna MP, Myers EW, Nickerson E, Nobile JR, Plant R, Puc BP, Ronan MT, Roth GT, Sarkis GJ, Simons JF, Simpson JW, Srinivasan M, Tartaro KR, Tomasz A, Vogt KA, et al 2005 Genome sequencing in microfabricated high-density picolitre reactors. Nature 437:376–380. doi:10.1038/nature03959. 16056220 PMC1464427

[3] Wu YW, Simmons BA, Singer SW 2016 MaxBin 2.0: an automated binning algorithm to recover genomes from multiple metagenomic datasets. Bioinformatics 32:605–607. doi:10.1093/bioinformatics/btv638. 26515820

[4] Huson DH, Auch AF, Qi J, Schuster SC 2007 MegaN analysis of metagenomic data. Genome Res 17:377–386. doi:10.1101/gr.5969107. 17255551 PMC1800929

[5] Rissman AI, Mau B, Biehl BS, Darling AE, Glasner JD, Perna NT 2009 Reordering contigs of draft genomes using the Mauve Aligner. Bioinformatics 25:2071–2073. doi:10.1093/bioinformatics/btp356. 19515959 PMC2723005

[6] Kearse M, Moir R, Wilson A, Stones-Havas S, Cheung M, Sturrock S, Buxton S, Cooper A, Markowitz S, Duran C, Thierer T, Ashton B, Meintjes P, Drummond A 2012 Geneious Basic: an integrated and extendable desktop software platform for the organization and analysis of sequence data. Bioinformatics 28:1647–1649. doi:10.1093/bioinformatics/bts199. 22543367 PMC3371832

[7] Tatusova T, Dicuccio M, Badretdin A, Chetvernin V, Nawrocki EP, Zaslavsky L, Lomsadze A, Pruitt KD, Borodovsky M, Ostell J 2016 NCBI Prokaryotic Genome Annotation Pipeline. Nucleic Acids Res 44:6614–6624. doi:10.1093/nar/gkw569. 27342282 PMC5001611

